# Patient experience and resource use analysis of balloon-retained vs. disc-retained gastro-jejunal tubes in children

**DOI:** 10.1007/s00383-026-06680-4

**Published:** 2026-10-15

**Authors:** Muhammad Saad Ali, Saifullah Khan, Olugbenga Michael Aworanti

**Affiliations:** 1 Department of Paediatric Surgery, Leeds Childrens Hospital, Leeds Teaching Hospitals NHS Trust, Leeds, LS1 3EX UK; 2 Leeds Institute of Medical Research, School of Medicine, Faculty of Medicine and Health, University of Leeds, Leeds, LS9 7TF UK

**Keywords:** Gastrojejunal feeding, Balloon-retained gastro-jejunostomy, Disc-retained gastro-jejunostomy, Patient-reported outcomes, Patient experience, Health economics

## Abstract

**Purpose:**

Building on our previous work demonstrating that balloon-retained gastro-jejunostomy tubes (BGJ) avoid complications unique to disc-retained devices but require more frequent replacement, we evaluated caregiver experience and healthcare resource use to better inform tube selection in children.

**Methods:**

Families whose children had experienced both Freka® percutaneous endoscopic gastro-jejunostomy (FPEGJ) and BGJ completed a paired questionnaire assessing nine experience domains (0–5 Likert-type scales) with free-text comments. Scores were compared using the Wilcoxon signed-rank test and comments underwent thematic analysis. A 24-month NHS resource-use model compared staged FPEGJ followed by planned BGJ conversion with primary laparoscopic BGJ insertion using 2026/27 NHS Payment Scheme tariffs.

**Results:**

Twelve of thirteen eligible families responded(92.3%). Nine(75%) preferred BGJ and three had no preference. No questionnaire domain differed significantly, although BGJ showed trends towards greater comfort and easier daily care, while FPEGJ showed a trend towards less leakage. Families favoured BGJ because of its lower profile, avoidance of general anaesthesia (GA) for replacement and avoidance of buried bumper syndrome. Standard staged FPEGJ treatment cost £4,192 over 24 months compared with £6,024 for primary laparoscopic BGJ, saving £1,831(30.4%). Even with laparoscopic assistance, FPEGJ remained 22.1% less expensive. Routine BGJ exchanges were the principal cost driver.

**Conclusions:**

A staged FPEGJ-to-BGJ strategy appears to provide the best balance between caregiver experience and NHS resource use. However, disc-retained devices commit children to at least one future GA, the associated burden of which is not fully captured by cost analysis. BGJ with reliable 12-month durability may further reduce procedural burden and healthcare costs.

**Supplementary Information:**

The online version contains supplementary material available at https://doi.org/10.1007/s00383-026-06680-4.

## Introduction

Jejunal feeding is increasingly recognised as an effective long-term enteral feeding strategy, particularly for neurologically impaired children with gastro-oesophageal reflux and gastrointestinal dystonia [1, 2]. We recently reported the first comparative analysis of the two principal gastro-jejunostomy (GJ) tube systems used to achieve jejunal feeding in children [3]. That study demonstrated that Freka^®^ percutaneous endoscopic gastro-jejunostomy tubes (FPEGJ) (Fresenius Kabi, Bad Homburg, Germany) [4] with solid internal discs were associated with fewer routine tube exchanges and were particularly advantageous in smaller infants, although they carried a risk of significant complications, including buried bumper syndrome, distal migration, and gastrocolic fistula. In contrast, balloon-retained gastro-jejunostomy tubes (BGJ) [5, 6] appeared to reduce major morbidity without increasing the risk of acute tube displacement.

Clinical outcomes alone, however, do not determine the optimal GJ tube. Family experience and healthcare resource utilisation are equally important in guiding practice. We therefore surveyed families whose children had experienced both tube types to determine their preferences and performed a 24-month National Health Service (NHS) resource-use analysis comparing primary laparoscopic BGJ insertion with primary FPEGJ insertion followed by planned conversion to BGJ. By integrating clinical outcomes, patient experience and healthcare resource utilisation, we aimed to provide a comprehensive evidence base to inform clinicians selecting the most appropriate GJ tube for children requiring long-term jejunal feeding.

## Methods

We conducted a mixed-methods evaluation comprising (1) a prospective assessment of family-reported experience with FPEGJ and BGJ tubes, and (2) a 24-month NHS resource use and cost comparison of the two clinical pathways. All clinical event rates (routine and acute tube changes, buried bumper, gastrocolic fistula) were taken from our previously published retrospective cohort study of 104 GJ tube episodes [3].

###  Methods: patient experience study

#### Participants

Families were eligible if their child had used both a FPEGJ and a BGJ within the preceding 48 months, with at least 6 months’ experience of each device, thereby minimising recall bias. Eligible families were identified from our institutional GJ tube database, and all were approached by telephone, with up to two follow-up attempts for non-response. Consistent with local clinical practice, most children experienced the devices sequentially, transitioning from FPEGJ to BGJ. Children who underwent reverse conversion were excluded, as this occurred infrequently and usually reflected specific clinical indications rather than routine management. We aimed to recruit approximately 20 families, anticipating thematic saturation.

#### Survey

In the absence of a validated questionnaire specifically comparing caregiver experience of these two GJ tube types, a structured questionnaire was developed for this study. Families rated their experience of FPEGJ and BGJ separately across nine domains using 0–5 Likert-type scales, with higher scores indicating a more favourable experience:

- Comfort.
- Leakage.
- Ease of daily care.
- Feeding reliability.
- Caregiver burden.
- Impact on sleep.
- Anxiety about tube dislodgement.
- Burden of hospital visits (Emergency Department, Interventional Radiology or general anaesthesia).
- Overall experience.

Families were also asked which device they would prefer to use over the following 12 months and to explain the reasons for their preference.

#### Analysis

Domain scores for FPEGJ and BGJ were compared using the Wilcoxon signed-rank test, with statistical analyses performed using Python (Python Software Foundation). Results are presented as median (interquartile range [IQR]). P values < 0.05 were considered statistically significant. Given the small sample size and the largely fixed sequence of device exposure, analyses were considered exploratory and interpreted as within-family comparisons rather than a causal assessment of device superiority. Qualitative free-text responses were analysed thematically to identify recurring themes and to contextualise the quantitative findings.

### Methods: resource use and cost analysis

A 24-month resource-use and cost analysis was performed from the NHS England provider/commissioner perspective comparing FPEGJ and BGJ. Acute tube change rates were derived from our previous comparative study (0.06/month for FPEGJ and 0.05/month for BGJ), equating to approximately one acute tube change over a 24-month period [3].

Two clinical pathways were modelled. The FPEGJ pathway comprised primary FPEGJ insertion under general anaesthesia (GA), one planned fluoroscopic jejunal-limb exchange at 12 months, one acute tube change and planned conversion to BGJ under GA after approximately 24 months or longer if an acute change occurred. The BGJ pathway comprised primary laparoscopic BGJ insertion, four routine 6-monthly fluoroscopic exchanges and one acute tube change. A secondary analysis incorporated laparoscopic-assisted FPEGJ insertion together with operative management of buried bumper syndrome.

Procedural costs were derived from the NHS England 2026/27 National Health Service Payment Scheme (NHSPS) using Healthcare Resource Group (HRG)-aligned tariffs, primarily the combined day-case/ordinary elective spell tariff category, which reflects planned elective procedural activity and includes standard inpatient resource allocation before excess bed-day payments apply [7]. Device acquisition costs were obtained from NHS Supply Chain procurement pricing [8].

Primary FPEGJ insertion was costed using HRG FE12B (“Endoscopic insertion of gastrostomy tube, 18 years and under”; £1974), with separate addition of Freka^®^ PEG (£119.44 including value-added tax [VAT]) and jejunal extension (£100.78 including VAT) [8], resulting in a total insertion cost of £2,194.22. Planned and acute fluoroscopic jejunal-limb exchanges were costed using outpatient HRG YF01B (“Radiological insertion of gastrostomy tube, 18 years and under”; £296), with replacement jejunal extension costs added separately, giving £396.78 per exchange. Conversion from FPEGJ to BGJ was costed using HRG FE13Z (“Endoscopic insertion of gastrojejunostomy or jejunostomy tube”; £860) with addition of the mean BGJ device cost of £344.55 including VAT, derived from Avanos MIC^®^ GJ (Avanos Medical, Alpharetta, Georgia, USA) (£354.37 including VAT) and Avanos MIC-KEY^®^ GJ (£334.73 including VAT) [8], resulting in a total conversion cost of £1,204.55 [7, 8].

Primary laparoscopic BGJ insertion was costed using HRG FE12B (£1974) with an additional laparoscopic resource adjustment equivalent to 25% of HRG MA10Z (£502.50,“Minor laparoscopic or endoscopic upper genital tract procedures”), reflecting the incremental operative resource associated with laparoscopic gastric fixation while avoiding double-counting of theatre and anaesthetic costs already included within FE12B [7]. Addition of the mean BGJ device cost resulted in a total insertion cost of £2,821.05. Routine and acute BGJ exchanges were costed using outpatient HRG YF01B (£296) together with the mean BGJ device cost, giving £640.55 per exchange.

Laparoscopic-assisted FPEGJ insertion was modelled using the same MA10Z-derived laparoscopic resource adjustment, giving a total insertion cost of £2,696.72. Based on results from our previous study, a 5.4% probability of operative buried bumper retrieval requiring laparoscopic/laparotomy intervention was incorporated into the FPEGJ pathway using an additional procedural cost equivalent to the laparoscopic resource adjustment (£502.50).

Minor complications without clearly attributable procedural resource utilisation were excluded. Inpatient length of stay was not analysed separately because NHSPS tariffs already incorporate up to 5 inpatient days and admission duration was considered predominantly related to underlying patient comorbidity rather than tube type.

## Results

### Results: patient experience study

Thirteen families met the predefined eligibility criteria. Twelve families completed the questionnaire, giving a response rate of 92.3%, while one family declined participation.

Across the nine domains evaluated, median scores for FPEGJ and BGJ were generally similar, with no statistically significant paired differences identified using the Wilcoxon signed-rank test (Table 1). There was a trend towards higher BGJ scores for comfort and ease of daily care while FPEGJ trended towards less issues with leakage, although these differences did not reach statistical significance.

Despite the absence of significant differences in individual domain scores, overall device preference favoured BGJ. Nine of 12 families (75.0%) stated that they would choose a BGJ if their child required jejunal feeding for the next 12 months, while three families (25.0%) reported no preference. No family selected FPEGJ as their preferred device.

Qualitative responses provided important context for the quantitative findings (Table 2). Families who preferred BGJ most commonly emphasised practical advantages, particularly avoidance of GA, simpler radiological tube replacement and improved tube reliability. Several respondents also cited the lower-profile design of the balloon-retained tube, reporting less concern about accidental pulling or dislodgement, while one family identified previous buried bumper syndrome as the principal reason for preferring BGJ.

Families expressing no clear preference generally recognised distinct advantages and disadvantages of both tube types and felt that device choice should be individualised.

**Table 1:** Parent-reported experience scores comparing Freka^®^ percutaneous endoscopic gastro-jejunostomy (FPEGJ) and balloon-retained gastro-jejunostomy (BGJ) tubes

Domain	FPEGJ median (IQR)	BGJ median (IQR)	*p* value
Comfort	2 (2–3)	4 (3.8–5)	0.087
Leakage	4.5 (2.8–5)	3.5 (2–5)	0.469
Ease of daily care	3 (2.5–4.3)	5 (3.8–5)	0.082
Feeding reliability	3.5 (3–4.3)	4 (4–5)	0.313
Carer burden	5 (3–5)	5 (4–5)	0.375
Sleep impact	5 (4–5)	5 (3.8–5)	0.750
Anxiety about dislodgement	2.5 (1.8–3.5)	3 (2–3)	1.000
Burden of hospital visits	2 (1–3.3)	2 (1.8–3)	0.766
Overall experience	3 (2–4)	3.5 (2.8–5)	0.240

Data are presented as median (interquartile range [IQR]). P values were calculated using the Wilcoxon signed-rank test. Higher scores indicate a more favourable experience (0 = worst, 5 = best)

**Table 2:** Thematic analysis of parent-reported reasons for tube preference following experience with both Freka^®^ percutaneous endoscopic gastro-jejunostomy (FPEGJ) and balloon-retained gastro-jejunostomy (BGJ) tubes

Theme	*n*/12 (%)	Example comment
Avoidance of general anaesthesia (GA)/surgery	5 (41.7%)	“Avoids the risks of GA”, “does not require GA”, “less invasive”
Easier replacement by Interventional Radiology	3 (25.0%)	“Easily replaced by a radiologist”, “appointments easier to arrange”
Fewer blockages/better tube reliability	3 (25.0%)	“Fewer blockages”, “stays securely in place”
External Freka tube dangles/easier to catch or pull	2 (16.7%)	“Freka hangs outside the body”, “tends to dangle”
Previous buried bumper syndrome	1 (8.3%)	Previous buried bumper experience
Less leakage	1 (8.3%)	“BGJ has fewer leakages”
No clear preference/both have advantages and disadvantages	3 (25.0%)	“Both tubes have issues”, “not sure which is better”

Responses were coded into recurring themes.Individual responses could contribute to more than one theme; therefore,percentages do not total 100%

### Results: resource-use and cost analysis (Table 3)

The base-case 24-month FPEGJ pathway without laparoscopic assistance resulted in a total estimated NHS cost of £4,192.33 per patient. This comprised initial FPEGJ insertion under GA, one planned fluoroscopic jejunal-limb exchange, one acute unplanned tube change and final conversion to a BGJ under GA.

Routine BGJ maintenance represented the largest contributor to overall pathway cost. Each fluoroscopic BGJ exchange was costed at £640.55, and four routine exchanges over the 24-month study period (6, 12, 18 and 24 months) resulted in a cumulative planned maintenance cost of £2,562.20. One additional acute BGJ exchange was modelled, adding a further £640.55. Consequently, the total 24-month laparoscopic BGJ pathway cost, incorporating primary laparoscopic BGJ insertion under GA, four routine fluoroscopic tube exchanges and one acute unplanned tube change, was £6,023.80.

Compared with primary laparoscopic BGJ insertion, the standard FPEGJ pathway reduced NHS costs by £1,831.47 per patient over 24 months, representing a 30.4% cost saving.

Previous work demonstrated a 5.4% incidence of buried bumper syndrome requiring laparoscopic/laparotomy retrieval and a 1.8% incidence of gastrocolic fistula following FPEGJ insertion. A secondary analysis therefore incorporated laparoscopic assistance during FPEGJ insertion, representing cases where laparoscopy was used to minimise the risk of gastrocolic fistula or operative management of buried bumper complications. The total laparoscopic-assisted FPEGJ pathway cost over 24 months was £4,694.83.

Compared with the laparoscopic BGJ pathway, the laparoscopic-assisted FPEGJ pathway reduced NHS costs by £1,328.97 per patient over 24 months, representing a 22.1% cost saving.

If laparoscopic assistance was utilised during FPEGJ insertion and operative laparoscopy/laparotomy was also subsequently required to retrieve a buried bumper during tube conversion, the total pathway cost increased to £5,197.33.

Even under this scenario, the laparoscopic BGJ pathway remained £826.47 more expensive than the FPEGJ pathway, a 15.9% increase in overall NHS costs.

**Table 3:** Estimated 24-month National Health Service (NHS) costs for modelled gastro-jejunostomy treatment pathways

24 Month Pathway + 1 acute change	Total cost
Standard FPEGJ insertion	£4,192.33
Laparoscopic-assisted FPEGJ insertion	£4,694.83
Laparoscopic-assisted FPEGJ + operative buried bumper retrieval	£5,197.33
Laparoscopically inserted BGJ	£6,023.80

FPEGJ, Freka® percutaneous endoscopic gastro-jejunostomy; BGJ, balloon-retained gastro-jejunostomy. Costs represent the estimated total NHS expenditure per patient over 24 months and include one acute unplanned tube change in each pathway

## Discussion

This study extends our previous comparative evaluation of GJ tubes [3] by integrating patient experience with healthcare resource utilisation to inform selection of the most appropriate enteral access strategy for children requiring long-term jejunal feeding. While our earlier work demonstrated that FPEGJ tubes required fewer routine interventions but were associated with important complications, the present study addresses the practical question faced by clinicians and families: which treatment pathway provides the greatest overall value when patient preference, procedural burden and healthcare costs are considered?

Although quantitative questionnaire scores did not demonstrate statistically significant differences between devices, three-quarters of families stated that they would choose a BGJ tube if their child required jejunal feeding again. The qualitative responses provide important context for this apparent discrepancy. Rather than focusing on individual symptoms such as comfort or leakage, families consistently prioritised practical aspects of long-term care, particularly avoiding repeated GA, simpler replacement by interventional radiology, improved mechanical reliability and the lower-profile design of BGJ tubes. The trend towards lower leakage scores with FPEGJ may reflect the more secure seal provided by the disc-retained internal bumper, whereas lower ease-of-care scores likely relate to the need for regular advancement of the tube to prevent buried bumper syndrome. Notably, one family that had previously experienced buried bumper syndrome preferred BGJ for future treatment despite reporting a greater hospital visit burden and poorer overall experience with BGJ, suggesting that the morbidity associated with buried bumper syndrome [9, 10] including pain, inability to use the tube, local abscess formation, and the need for urgent operative intervention, may outweigh concerns regarding relatively minor day-to-day complications such as leakage.

The resource-use analysis produced equally important findings. The staged FPEGJ pathway was substantially less expensive than primary laparoscopic BGJ insertion over 24 months, reducing NHS costs by approximately 30% (£1,831 per patient). Even when laparoscopic assistance during FPEGJ insertion was modelled, the pathway remained approximately 22% less expensive than primary laparoscopic BGJ insertion. Furthermore, incorporating the additional cost of operative buried bumper retrieval did not eliminate this economic advantage. However, our model almost certainly underestimates the true burden associated with buried bumper syndrome, as it does not account for the additional outpatient assessments, emergency presentations, unplanned admissions, disruption to enteral feeding, caregiver anxiety or the urgency of arranging operative intervention to restore safe jejunal feeding [9, 10].

Irrespective of cost, placement of a disc-retained device commits the child to at least one subsequent procedure under GA. The value of avoiding this additional intervention extends beyond direct healthcare expenditure and is difficult to quantify, encompassing the medical risks of repeated anaesthesia, family disruption, caregiver anxiety and the cumulative burden of hospital attendance for children with complex medical needs.

The principal driver of BGJ pathway costs was the requirement for four routine fluoroscopic tube exchanges over 24 months together with the relatively high acquisition cost of the balloon-retained device. Our analysis used the mean procurement cost of the Avanos MIC^®^ and MIC-KEY^®^ gastro-jejunostomy tubes (£344.55 including VAT), representing current local NHS procurement prices. Use of alternative devices, such as the AMT G-Jet^®^ (Applied Medical Technology Inc., Brecksville, Ohio, USA) [6], which currently costs £533.56 including VAT [8], would further increase the overall cost of the BGJ pathway. At present, there is no published evidence demonstrating improved durability or longer exchange intervals for this device to justify its substantially higher acquisition cost.

The optimal interval for routine BGJ replacement remains uncertain. In our institution, balloon-retained tubes are routinely exchanged every six months following a previous departmental audit demonstrating fewer balloon-related complications when tubes were not left in situ beyond this period. Similar replacement schedules have been reported by other paediatric centres [11] whereas others advocate intervals of 6–12 months or have questioned whether routine replacement is required at all [12–14]. Following completion of this study, Avanos advised that balloon longevity is highly variable and typically ranges from 1 to 8 months, depending on balloon inflation volume, gastric pH and routine tube care [15]. These observations have prompted consideration of extending our routine exchange interval to eight months with prospective audit of clinical outcomes. More broadly, the requirement for frequent routine exchanges represents a substantial and potentially avoidable burden on families, interventional radiology services and NHS resources. Future device innovation should therefore focus not only on tube design but also on improving balloon durability [16], with a reliable 12-month lifespan representing a potentially important opportunity to reduce healthcare costs, radiation exposure and procedural burden without compromising patient safety.

Our economic findings complement those of the only previous paediatric economic evaluation comparing disc-retained and balloon-retained gastro-jejunostomy tubes by Rose and Patel 2021 [14]. Using a fundamentally different methodology based on single-centre bottom-up micro-costing, they reported remarkably similar procedural costs to those generated by our HRG-based model, supporting the face validity of the present costing estimates. Their study concluded that prolonged maintenance of disc-retained tubes resulted in a significantly higher cost per day despite fewer interventions, largely because complete tube replacement required hospital admission and GA. In contrast, our study evaluated a planned staged treatment strategy in which FPEGJ serves as temporary enteral access before elective conversion to a balloon-retained tube after approximately two years. Consequently, our analysis avoids the repeated GA costs associated with long-term maintenance of a disc-retained device while also demonstrating that immediate primary laparoscopic BGJ insertion represents the more expensive treatment pathway. The two studies therefore evaluate different clinical strategies but reach complementary conclusions: prolonged maintenance of FPEGJ is economically undesirable, whereas planned conversion to BGJ appears to balance safety, patient experience and healthcare resource utilisation.

The present findings should not be interpreted as supporting one tube for every child. Our previous work demonstrated that FPEGJ remains particularly valuable in smaller infants, where its narrower calibre may reduce the risk of bowel perforation associated with larger balloon-retained devices, while BGJ tubes avoid complications unique to solid internal bumpers such as buried bumper syndrome [3].

Rather than advocating one device over another, our findings support a staged approach that utilises the strengths of each tube at different stages of treatment. Initial FPEGJ insertion appears well suited to appropriately selected infants, providing a cost-effective means of establishing durable jejunal access with relatively few planned interventions. Elective conversion to a BGJ tube following maturation of the gastrostomy tract may subsequently reduce caregiver burden and avoid complications such as buried bumper syndrome. Whether the additional cost of primary laparoscopic BGJ insertion is justified by avoiding these complications and subsequent GA remains uncertain and is likely to depend on patient selection, local expertise and healthcare priorities. Furthermore, the development of more durable balloon-retained devices, together with extending routine BGJ replacement intervals, could substantially improve the cost-effectiveness of this strategy. In the future, the availability of narrow-calibre balloon-retained GJ tubes suitable for primary insertion in infants, such as the Micro G-JET^®^ low-profile gastro-jejunal tube (Applied Medical Technology Inc., Brecksville, Ohio, USA) [17], with demonstrated safety and prolonged maintenance intervals (for example, 12-monthly exchange), may represent the optimal long-term enteral-access strategy by avoiding both repeated GA in children with complex medical needs and complications unique to disc-retained devices.

Several limitations should be acknowledged. The patient-experience study was exploratory, involving a small number of families from a single centre using a locally developed, non-validated questionnaire. All children experienced the devices sequentially, introducing potential sequence bias and confounding from increasing age, clinical stability and caregiver familiarity over time. Recall bias and response bias are also possible despite restricting recruitment to families with relatively recent experience of both devices. Similarly, the economic analysis was model-based and reflects local clinical pathways and NHS England reimbursement tariffs, which may differ in other healthcare systems. Nevertheless, the model was derived from prospectively collected institutional outcome data, employed nationally applicable HRG tariffs, and showed close agreement with the only published paediatric micro-costing study [14], supporting its validity.

## Conclusion

When clinical outcomes, caregiver experience and healthcare resource utilisation are considered together, a staged treatment strategy of initial Freka^®^ percutaneous endoscopic gastro-jejunostomy (FPEGJ) followed by planned conversion to a balloon-retained gastro-jejunostomy (BGJ) currently appears to provide the best balance between safety, patient experience and NHS resource use. Although families consistently preferred BGJ because of its lower profile, avoidance of repeated GA and complications unique to disc-retained tubes, primary laparoscopic BGJ insertion remains substantially more expensive, largely because of the need for frequent routine exchanges. Conversely, use of a disc-retained device commits the child to at least one future procedure under GA, the burden of which extends beyond direct healthcare costs to include additional medical risk and caregiver anxiety.

Future innovation should therefore focus on improving balloon-retained GJ technology rather than disc-retained devices. The development of narrow-calibre balloon-retained GJ tubes suitable for primary insertion in infants, combined with durable balloons capable of reliably remaining in situ for 12 months or longer, has the potential to minimise GA, eliminate complications unique to disc-retained devices and substantially reduce healthcare costs associated with repeated routine tube replacement. Such advances may ultimately represent the optimal long-term enteral access strategy for children requiring jejunal feeding.

## Supplementary Information

Below is the link to the electronic supplementary material.

Supplementary Material 1

Supplementary Material 2

Supplementary Material 3

## Data Availability

No datasets were generated or analysed during the current study.
