# Supplementary material for: Patient experience and resource use analysis of balloon-retained vs. disc-retained gastro-jejunal tubes in children
Source: Pediatr Surg Int. 2026 Oct 15;42(1):445. doi: 10.1007/s00383-026-06680-4 (PMC13672259; doi:10.1007/s00383-026-06680-4)
Supplement: Supplementary file 1 [file 383_2026_6680_MOESM1_ESM.docx]

**Patient experience and Resource Use Analysis of Balloon-retained vs Disc-retained Gastro-Jejunal Tubes in Children.**

Authors:

1 Muhammad Saad Ali^a^

2 Saifullah Khan^a^

3 Olugbenga Michael Aworanti^a,b^

^a^Department of Paediatric Surgery, Leeds Childrens Hospital, Leeds Teaching Hospitals NHS Trust, Leeds LS1 3EX, UK

^b^Leeds Institute of Medical Research, School of Medicine, Faculty of Medicine and Health, University of Leeds, Leeds LS9 7TF, UK

corresponding author:

1 Muhammad Saad Ali (muhammad.ali75@nhs.net)

2 Olugbenga Michael Aworanti (olugbenga.aworanti@nhs.net)
