# Supplementary material for: Patient experience and resource use analysis of balloon-retained vs. disc-retained gastro-jejunal tubes in children
Source: Pediatr Surg Int. 2026 Oct 15;42(1):445. doi: 10.1007/s00383-026-06680-4 (PMC13672259; doi:10.1007/s00383-026-06680-4)
Supplement: Supplementary file 2 [file 383_2026_6680_MOESM2_ESM.docx]

1. Gastrojejunal feeding
2. Balloon-retained gastro-jejunostomy
3. Disc-retained gastro-jejunostomy
4. Patient-reported outcomes
5. Patient experience
6. Health economics
