# Supplementary material for: Patient experience and resource use analysis of balloon-retained vs. disc-retained gastro-jejunal tubes in children
Source: Pediatr Surg Int. 2026 Oct 15;42(1):445. doi: 10.1007/s00383-026-06680-4 (PMC13672259; doi:10.1007/s00383-026-06680-4)
Supplement: Supplementary file 3 [file 383_2026_6680_MOESM3_ESM.docx]

* Most families preferred balloon-retained gastro-jejunostomy tubes for long-term use.

* This preference reflected, a lower-profile device and avoidance of future general anaesthesia for routine change.

* A staged pathway beginning with Freka percutaneous endoscopic gastro-jejunostomy reduced 24-month healthcare costs by 30% compared with primary laparoscopic balloon-retained tube placement.

* The higher cost of balloon-retained tubes was driven mainly by frequent planned replacements.

* Smaller balloon-retained tubes with reliable 12-month durability could reduce general anaesthesia, complications and healthcare resource use.
